# MIR-99a and MIR-99b Modulate TGF-β Induced Epithelial to Mesenchymal Plasticity in Normal Murine Mammary Gland Cells

**DOI:** 10.1371/journal.pone.0031032

**Published:** 2012-01-27

**Authors:** Gianluca Turcatel, Nicole Rubin, Ahmed El-Hashash, David Warburton

**Affiliations:** Developmental Biology and Regenerative Medicine Program, Saban Research Institute of Children's Hospital Los Angeles, Los Angeles, California, United States of America; Wayne State University, United States of America

## Abstract

Epithelial to mesenchymal transition (EMT) is a key process during embryonic development and disease development and progression. During EMT, epithelial cells lose epithelial features and express mesenchymal cell markers, which correlate with increased cell migration and invasion. Transforming growth factor-β (TGF-β) is a multifunctional cytokine that induces EMT in multiple cell types. The TGF-β pathway is regulated by microRNAs (miRNAs), which are small non-coding RNAs regulating the translation of specific messenger RNAs.

Herein, we identified mir-99a and mir-99b as two novel TGF-β target miRNA genes, the expression of which increased during TGF-β induced EMT of NMUMG cells. Mir-99a and mir-99b inhibition decreased TGF-β activity by inhibiting SMAD3 phosphorylation, resulting in decreased migration and increased proliferation in response to TGF-β. However, mir-99a and mir-99b inhibition was insufficient to block TGF-β induced EMT of NMUMG cells.

Mir-99a and mir-99b over-expression in epithelial NMUMG cells resulted in increased proliferation, migration and fibronectin expression, while E-cadherin and ZO-1 expression were negatively regulated.

In conclusion, we identified mir-99a and mir-99b as two novel modulators of TGF-β pathway that alter SMAD3 phosphorylation, in turn altering cell migration and adhesion of mesenchymal NMUMG cells. The effect of mir-99a and mir-99b over-expression on NMUMUG proliferation is dependent upon the epithelial or mesenchymal status of the cells. Our study suggests that mir-99a and mir-99b may function as modulators within a complex network of factors regulating TGF-β induced breast epithelial to mesenchymal transition, as well as proliferation and migration of breast cancer cells, providing a possible target for future translationally oriented studies in this area.

## Introduction

Epithelial to mesenchymal transition (EMT) is a complex process, which involves cytoskeletal remodeling and cell–cell and cell–matrix adhesion as well as transcriptional regulation, leading to the transition from a polarized epithelial phenotype to an elongated fibroblast-like phenotype. TGF-β is a secreted cytokine that regulates a variety of processes in development and cancer including epithelial to mesenchymal transition [1], [2]. The TGF-β pathway cross-talks with other important molecular pathways, such as Wnt, and also acts thorough mTOR, which is activated through phosphorylation by TGF-β itself. In turn mTOR negatively regulates TGF-β signaling through SMAD3 inhibition.

Comparison of the genomes of different species has shown that a large proportion of the genome is devoted to controlling gene transcription. MicroRNAs (mirnas) are single stranded RNAs, 19–25 nucleotides in length that are generated from endogenous hairpin shaped transcripts [3]. Mirnas are regulatory genes that inhibit gene expression of specific target genes, primarily by binding to the 3′ UTR of the specific mRNA [4]–[7]. They have important roles in many biological processes such as cell proliferation, differentiation and embryonic development as well as in the development and progression of diseases [8]–[11].

Dicer is the key enzyme involved in mirna biogenesis and it also plays a direct role in the process of EMT. Down-regulation of DICER by miR-103/107 induces EMT of NMUMG cells, which results in enhanced cell migration and metastatic properties [12]. Likewise, the maturation and processing of mirna has been directly connected to the TGF-β pathway [13].

Mirnas are already known to be key regulators of the TGF-β pathway [14]–[18]. For example, the mir-200 family of mirnas is specifically down-regulated by TGF-β during EMT in normal mouse mammary gland (NMUMG) cells, whereas up-regulation of mir-200s in epithelial phase NMUMG cells completely abrogates TGF-β pathway signaling and thus TGF-β mediated stimulation of EMT [19]–[22]. Additionally, Mir-155 is a downstream mirna of the TGF-β pathway that can modulate epithelial cell plasticity [23]. mTOR is a target of mir-99a and mir-99b [24], [25]. By targeting mTOR, mir-99a and mir-99b inhibit proliferation of c-Src-transformed cells and prostate cancer cells. [24], [25]. However, Li X et al. (2011) reported that mir-99a and mir-99b are over-expressed in gastric carcinoma [26], which indicates that mir-99a and mir-99b may also act as oncomirs in different cell types. Although mTOR plays a key role in cell proliferation and differentiation, its inhibition with rapamycin does not affect proliferation in some cell lines *in vitro* [27], [28]. Herein, we focused on determining whether the mir-99a and mir-99b family of mirnas play a functional role in modulating the TGF-β pathway and their role on cell proliferation in epithelial NMUMG cells, which are insensitive to rapamycin, versus mesenchymal NMUMG cells that are instead rapamycin sensitive [28].

In our study we have identified mir-99a and mir-99b as two novel downstream mirnas of the TGF-β pathway. The expression of mir-99a and mir-99b was stimulated by TGF-β during TGF-β induced EMT in NMUMG cells. The blockade of mir-99a and mir-99b with LNA-knockdown probes inhibited TGF-β autocrine activity in NMUMG cells through inhibition of Smad3 phosphorylation and consequently inhibited cell migration, increased cell proliferation, yet failed to completely arrest EMT. On the other hand, up-regulation of mir-99a and mir-99b in NMUMG cells resulted in down-regulation of E-cadherin and ZO-1, together with increased cell migration and proliferation. We have validated multiple targets of mir-99a and mir-99b that are known to be involved in cell proliferation, and differentiation, as well as chromatin remodeling. Some important mir-99a and mir-99b effects such as E-cadherin and ZO-1 down-regulation could be replicated by mTOR down-regulation using a specific sirna. Thus, mTOR may be considered as a main functional target of mir-99a and mir-99b among a rather broad network of targets modulating different aspects of cellular function. In particular, by negatively modulating TGF-β pathway signaling and therefore epithelial and mesenchymal cell plasticity, we speculate that mir-99a and mir-99b may prove to be critical modulators of cancer development and progression. In addition, we speculate that by down-regulating the mTOR gene, these mirnas could perhaps counteract the over-activation of the mTOR pathway that is seen in diseases such as Lymphangioleiomyomatosis (LAM) and certain cancers.

## Materials and Methods

### Cell lines

HELA, NMUMG, 4T1 cells were purchased from ATCC and maintained in DMEM, 10% FBS (Gibco/Invitrogen, Carlsbad, CA) and 1% antibiotics (Penicillin, Streptomycin) (Gibco/Invitrogen, Carlsbad, CA). NMuMG cells media were supplemented with insulin (10 µg/ml) (Sigma-Aldrich, St. Louis, MO). 4T1 cells were cultured with RPMI-1640 supplemented with 10% FBS.

### CDNA retrotranscription and RT Real Time PCR

RNA was extracted from cell culture pellets using TRIZOL reagent. 1 µg of RNA was retro-transcribed using the SuperScript II reverse transcriptase kit (Invitrogen, Carlsbad, CA). The cDNA was amplified with TaqMan (Roche,) in the presence of gene specific primers and probes (Operon, Huntsville, AL) as indicated. The primers sequence used is reported in the appendix (Table 1).

**Table 1:** Primers used for real time RT-PCR.

snai-1-s	tggaaaggccttctctaggc
snai-1-as	tcagcaaaagcacggttg
sip1-s	tcactaatccgacagcttgc
sip1-as	gggactctttcgtcatctttactg
slug-s	tgcaagatctgtggcaagg
slug-as	cagtgagggcaagagaaagg
fibronectin-s	ccctgacactggagtgcttac
fibronectin-as	ccgttcgtgggggtagtag
rRNA 18s-s	aaatcagttatggttcctttggtc
rRNA 18s-as	gctctagaattaccacagttatccaa

### Micrornas retro-transcription and Real Time PCR

For mirnas quantification, TaqMan microRNAs Assay was used. For the retro-transcription 10 ng of total RNA was used and retro-transcribed in 7.5 µl total volume reactions containing: 1.5 µl mirna specific primer (Applied Biosystems), 10 units of RNAase inhibitor, and 25 units of multiscribe reverse retro-transcriptase (Chen et al., 2005). Quantitative real-time PCR was carried out using the Roche Light Cycler 480 and the Light Cycler TaqMan Master Mix. Real Time PCR conditions were as follows: 95°C for 10 minutes, 45 cycles: 95°C for 10 seconds, 60°C for 30 seconds. Mirnas specific primer and probe were supplied by Applied Biosystems.

### Luciferase plasmids design

To confirm mir-99a and mir99b targets, a luciferase assay was used. Part of the wild type 3′UTR (WT-3′-UTR) and mutated 3′UTR (MUT-3′UTR) of the hypothetical target gene messenger RNA, containing the putative mir-99a and mir-99b binding sites was amplified by PCR and inserted downstream of a luciferase reporter gene in a PGL4.13 plasmid (Promega, San Luis Obispo, CA). The mutated 3′UTR (MUT-3′-UTR) was obtained by inserting 3 point mutations in the mir-99a and mir-99b binding site, thus destroying the putative mirna/mRNA interaction. To create the 3′-UTR-MUT, a PCR approach was used using specific primers covering the mir-99a and mir-99b binding sites bearing the mutated bases. The sequence of the primers used for the 3′UTRs cloning is reported in appendix (Table 2).

**Table 2:** Primers used for 3′UTR cloning.

Gene name	Forward and reverse primers	Size of the 3′UTR
mTOR	For 5′-CCTTGTCTGTGCTTCCAGTG-3′	382 bp
(NM_020009.1)	Rev 5′-ACGGGTGAGGTAACAGGATG-3′	
PAM	For 5′-CAGATCCTTTGTCTATGGAGAGG-3′	530 bp
(NM_207215.2)	Rev 5′-TACAGGACAAACATTGATAGCTTTA-3′	
CDK7	For 5′-GACGCACAATGGACAGTTTCAC-3′	156 bp
(NM_009874.3)	Rev 5′-GAATTGTAAAATACATTTAATAAAAAT-3′	
EPC2	For 5′-GGAAATTATGCATCTAGCACATT-3′	380 bp
(NM_172663)	Rev 5′-CATTCTGAAACACCACTACACG-3′	
PHOX2B	For 5′-TGCGCAGTTTAGACATCTCTGT-3′	487 bp
( NM_008888.2)	Rev 5′-CAAGTTAATGGTACACTCTTGGAAAA-3′	
CYP26B1	For 5′-TTGCCCCTGTCCCATATTTA-3′	807 bp
(NM_175475.2)	Rev 5′-CAGAACCCCAAACCCTTCC-3′	
FOXA1	For 5′-GACCGAATTCCAAAATCCCAAT-3′	249 bp
(NM_008259.3)	Rev 5′-GACAAATCACATCGATAAAAATTAACA-3′	
CALM	For 5′-TGAAGTAACATGTTGCATGTGG-3′	420 bp
(NM_007589.4)	Rev 5′-TTGGAAAACAAATATACAACTTGG-3′	
BAZ2A	For 5′-GCCCTGAACATGCTGCTT-3′	1360 bp
(NM_054078.2)	Rev 5′-TGCATAATATAAAGTCAATTCAAA-3′	
SMARCA5	For 5′-GCATTTTTGTCTTATAATCACTAACTG-3′	589 bp
(NM_053124.2)	Rev 5′-AAAGGCCATTCATCCAACAA-3′	
EPC2	For 5′-GGAAATTATGCATCTAGCACATT-3′	380 bp
(NM_172663)	Rev 5′-CATTCTGAAACACCACTACACG-3′	
CTDSPL	For 5′-CGTTCTTGCCACCACAAAC-3′	672 bp
(NM_133710.2)	Rev 5′-ATCGGTGACAGGTGGTGTG-3′	

### Luciferase assay

Hela cells were seed in 24 well plates (80000 cells per well) and the next day transfected with 300 ng of the WT or MUT 3′UTR luciferase reporter construct, together with a mir-99a, mir-99b or a scrambled precursor (final concentration 20 nM) (Dharmacon, Lafayette, CO, USA) 50 ng of the renilla plasmid PGL-6.43 (Promega, San Luis Obispo, CA) was co-transfected into each well and used as the transfection internal control. Cells were collected 48 h after transfection, and luciferase activity was measured with a dual-luciferase reporter assay (Promega, San Luis Obispo, CA). The luciferase activity was normalized on the Renilla luciferase activity and expressed as true ratio between the luciferase activity of the WT-3′UTR plasmid and the MUT-3′UTR plasmid. Every luciferase assay experiment was repeated at least three times.

### Adenoviral transduction

Adenoviral construct for wild type mTOR expression was obtained from Christopher Rhodes (University of Chicago), amplified in HEK-293A cells and used at MOI of 10. Western blot was used to confirm the up-regulation of mTOR protein [29], [30].

### Antibodies

MTOR antibodies were purchased from Cell Signaling, ACTIN antibody from MP Biomedicals, CYCLIN D1, SM-22-α, antibody from Santa Cruz Biotechnology, E-cadherin from BD transduction, SMARCA5 antibody from Bethyl laboratories, ZO-1 from invitrogen.

### Western blot

Cells were seeded at 50% confluence and the next day transfected with mir-99a, mir-99b or a scrambled mirna precursor (final concentration 80 nM) (Dharmacon) using lipofectamine 2000 (Gibco/Invitrogen, Carlsbad, CA). Fresh media was added 6 hour later. 72 hours post transfection cells were lysed with Ripa buffer (1XTBS, 1% Nonidet P-40, 0.5% sodium deoxycholate, 0.1% SDS, 0.004% sodium azide) (Sigma-Aldrich, St. Louis, MO), supplied with phosphatase and protease inhibitors (PMSF solution, sodium orthovanadate solution and protease inhibitor cocktail solution) (Sigma-Aldrich, St. Louis, MO). B-ACTIN protein amount was measured in each western blot experiment and used as loading control.

### Cytofluorimetry and cell cycle analysis

Cells were seeded in 6 well plates at 40% confluency and, the next day, transfected with the indicated siRNA or mirna (80 nM final concentration) using lipofectamine 2000 (Gibco/Invitrogen, Carlsbad, CA). Fresh media was added 6 hours after transfection. After 72 hours cells were trypsinized, washed with PBS and fixed in ice with cold 70%/30% ethanol/PBS solution for 30 minutes. Cells were then washed with PBS and resuspended in propidium iodium (20 µg/ml) solution containing RNAse (200 µg/ml) for 30′ at room temperature.

Cellular DNA content was determined with a FACScalibur (Becton Dickinson) instrument.

### Migration Assay: scratch assay

Cells were seed in 12 well plates at 50% confluency and the next day transfected with mir-99a, mir-99b or a scrambled mirna at a final concentration of 80 nM. Media was changed 6 hours later. 72 hours post transfection, cell layer was scratched with a p200 tip pipette. The healing process was followed for the next 24 hours. The ability of the cells to close the wound was expressed as true ratio between the area of the wound at 24 hours post scratch and the area of wound width right after the scratch was done. Wound area was determined with Photoshop software.

### Cells adhesion assay

NMUMG cells were seed on a 12 wells plate and the next day transfected with the indicated mirna inhibitor or control. 3 days later cells were trypsinized and the same number of cells for each sample was seeded into wells of 6 well plates. 3 hours later floating cells were removed by two PBS washes and the adherent cells were trypsinized and counted.

### SIRNAs

SIRNA for mTOR and control sirna were bought from Applied Biosystems and used at a final concentration of 20 nM. Lipofectamine 2000 (Invitrogen) was used for the transfection.

### Data analysis

Data are presented as means±SD. Statistical significance between control and experimental samples was calculated using Student's t-test. Western blots are representative of three independent experiments. Densitometry analysis was obtained using Image J software.

## Results

### Mir-99a and mir-99b expression increased during TGF-β induced EMT in NMUMG cells

**Figure 1:**
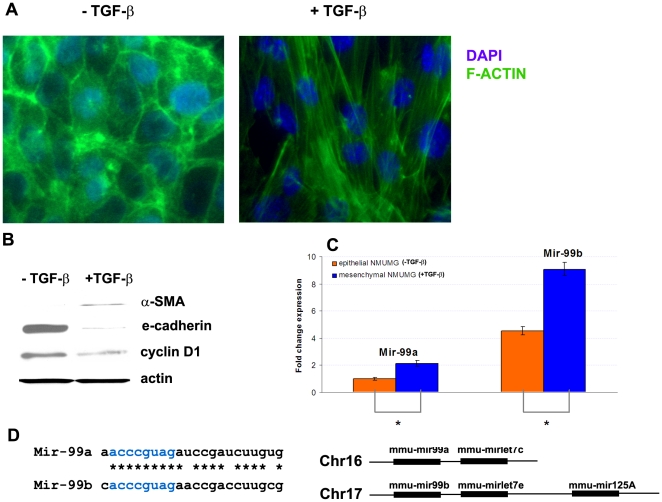
Mir-99a and mir-99b expression during TGF-β induced EMT of NMUMG cells. NMUMG cells were treated with TGF-β for 3 days and RNA was collected for mir-99a and mir-99b quantification. (A) F-actin staining shows changes of the morphology of NMUMG cells. (B) Quantification of mir-99a and mir-99b expression in TGF-β treated and untreated NMUMG cells (* = p<0.05). (C) Changes of E-cadherin, α-SMA, cyclin D1 expression during EMT of NMUMG cells. (D) Genomic organization of mir-99a and mir-99b loci.

When stimulated by TGF-β, NMUMG cells undergo EMT by showing visible morphological changes within 24 hours, and the EMT process can be considered complete in 3 days [31] (Figure 1A,B): mesenchymal NMUMG cells adopted a spindle-like shape which was correlated with actin reorganization, decreased E-cadherin expression and increased α-SMA expression. We used RT-Real Time PCR to determine whether the expression of mir-99a and mir-99b changes during TGF-β induced EMT in NMUMG cells. As shown in Figure 1C, mir-99a and mir-99b expression was higher in the mesenchymal versus the epithelial phase of NMUMG. Thus, we postulated that these mirnas may play specific functional roles in TGF-β stimulated EMT. Mir-99a and mir-99b share most of their nucleotide sequence and they are located in different chromosomes adjacent to the let-7 family of micrornas (Figure 1D), which suggests an evolutionary chromosome duplication.

### Mir-99a and mir-99b are required for normal TGF-β signaling in NMUMG cells

**Figure 2:**
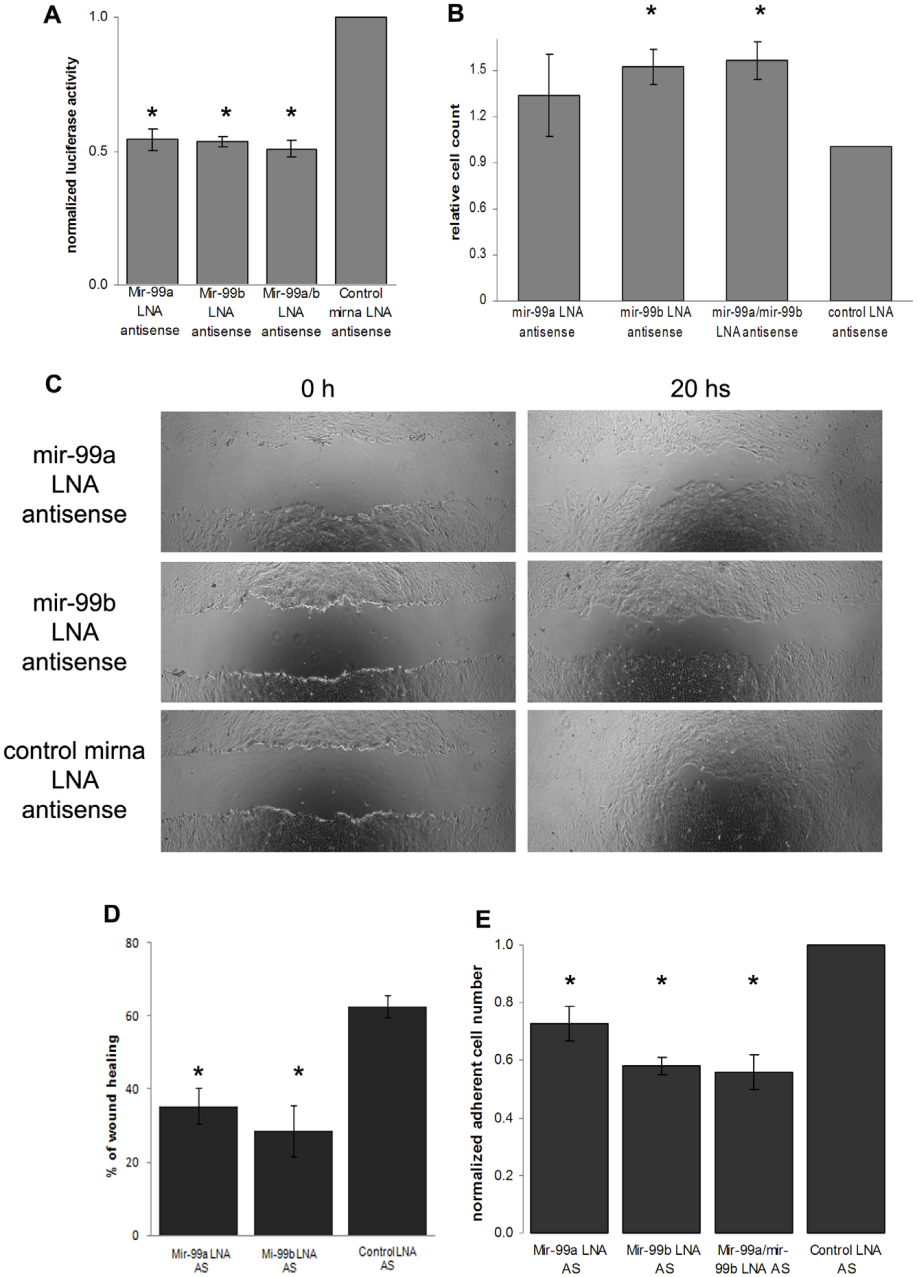
Role of mir-99a and mir-99b inhibition on TGF-β pathway. (A) Luciferase assay with a PAI-promoter luciferase reported plasmid shows that mir-99a and mir-99b blockade inhibits TGF-β pathway. (B) Inhibition of mir-99a and mir-99b with specific LNA antisense increases proliferation of mesenchymal phase NMUMG. (C,D) Inhibition of mir-99a and mir-99b inhibits migration of mesenchymal phase NMUMG cells and their adhesion abilities (E) (* = p<0.05).

As mentioned above, the expression of mir-99a and mir-99b increased during TGF-β induced EMT of NMUMG cells. Therefore, we used using specific LNA-probes for mir-99a and mir-99b to determine the effect of mir-99a and mir-99b blockade on the TGF-β signaling pathway and on the EMT process. NMUMG cells were transfected with the indicated LNA knock-down probes and two days later transfected with 3TP-lux plasmid, in which luciferase reporter gene expression is driven by a TGF-β sensitive promoter [32]. NMUMG cells were then incubated overnight with TGF-β and 24 hours later luciferase activity was quantified. The blockade of mir-99a and mir-99b with LNA-probe indeed inhibited the luciferase activity by about 50% (Figure 2A).

Next, we determined whether mir-99a and mir-99b blockade affected cell proliferation and migration in mesenchymal phase NMUMG cells. Cell proliferation of mesenchymal phase NMUMG cells was stimulated by inhibiting mir-99a and mir-99b with LNA-antisense probes (Figure 2B). TGF-β decreases proliferation of NMUMG cells but mir-99a and mir-99b blockade reversed the inhibitory effect of TGF-β on cells proliferation of NMUMG cells, supporting the hypothesis that mir-99a and mir-99b are necessary for normal TGF-β signaling.

Mir-99a and mir-99b inhibition also resulted in reduced cell migration (Figure 2C,D) and less efficient adhesion (Figure 2E) of mesenchymal phase NMUMG cells. Mir-99a and mir-99b blockade also inhibited TGF-β induced cell migration of human 4T1 cells (Figure S1A,B).

**Figure 3:**
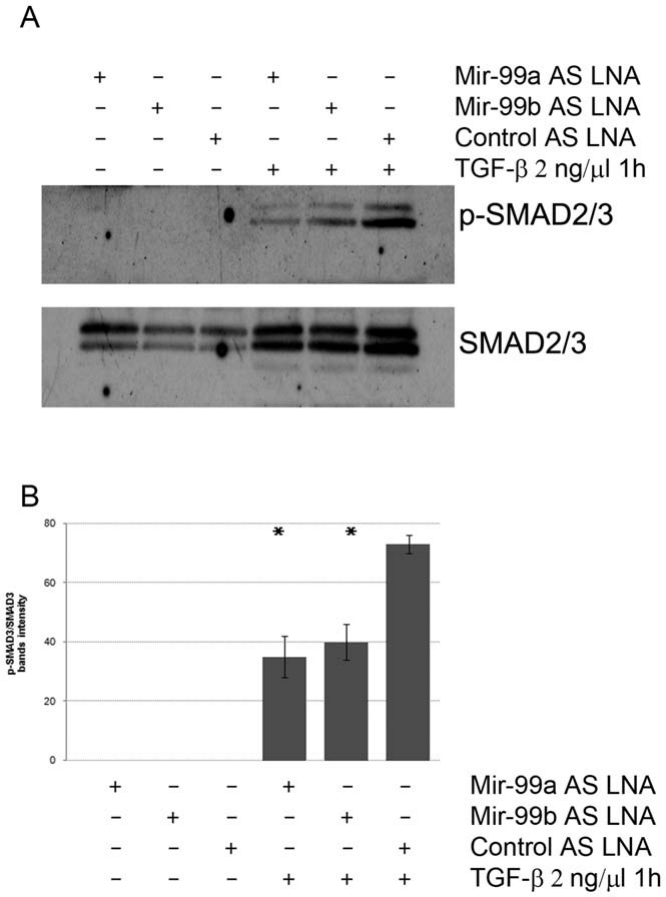
Mir-99a and mir-99b inhibition alters TGF-β induced SMAD3. (A,B) Mir-99a and mir-99b inhibition reduces TGF-β activity by inhibiting SMAD3 phosphorylation (* = p<0.05).

**Figure 4:**
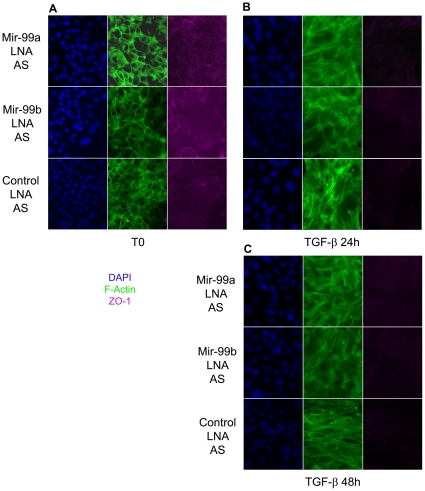
Mir-99a and mir-99b inhibition does not block TGF-β induced EMT of NMUMG cells. F- actin and ZO-1 staining of untreated (t0) (A) or TGF-β treated NMUMG cells for 24 h (B) or 48 h (C) shows that mir-99a and mir-99 inhibition does not block the transition of epithelial phase NMUMG cells into mesenchymal cells.

To determine whether the decreased TGF-β activity was accompanied with decreased SMAD3 phosphorylation, NMUMG cells were transfected with mir-99a, mir-99b or control LNA-probe, and pulsed 72 hours later with TGF-β recombinant protein. SMAD3 phosphorylation was then quantified by Western blot. Interestingly, SMAD3 phosphorylation by TGF-β was inhibited by mir-99a and mir-99b blockade (Figure 3A,B)., suggesting that mir-99a and mir-99b inhibition alters TGF-β pathway signaling by inhibiting phosphorylation of SMAD3. However, even though mir-99a and mir-99b blockade inhibited the TGF-β-SMAD3 pathway, TGF-β induced EMT of NMUMG cells was not apparently affected. Indeed, when cultured with TGF-β in the presence of mir-99a and mir-99b LNA-antisense probes, NMUMG cells still lost ZO-1 expression and assumed the morphological features of mesenchymal cells as indicated by the pattern of expression of filamentous actin (Figure 4A,B,C). Therefore, we concluded that mir-99a and mir-99b modulate downstream TGF-β signaling in NMUMG cells, affecting cell migration, adhesion and cell proliferation and we also concluded but that mir-99a and mir-99b are not needed for TGF-β induced EMT progression.

### Mir-99a and mir-99b over-expression increased cell motility and down-regulated E-cadherin and ZO-1 in epithelial NMUMG cells

Since the expression of mir-99a and mir-99b increased in NMUMG undergoing EMT (Figure 1C), and the blockade of mir-99a and mir-99b with LNA probes significantly affected mesenchymal phase NMUMG cell behavior, but did not fully arrest TGF-β induced EMT progression, we next determined whether the over-expression of mir-99a and mir-99b in epithelial phase NMUMG cells could induce their transition into mesenchymal cells.

**Figure 5:**
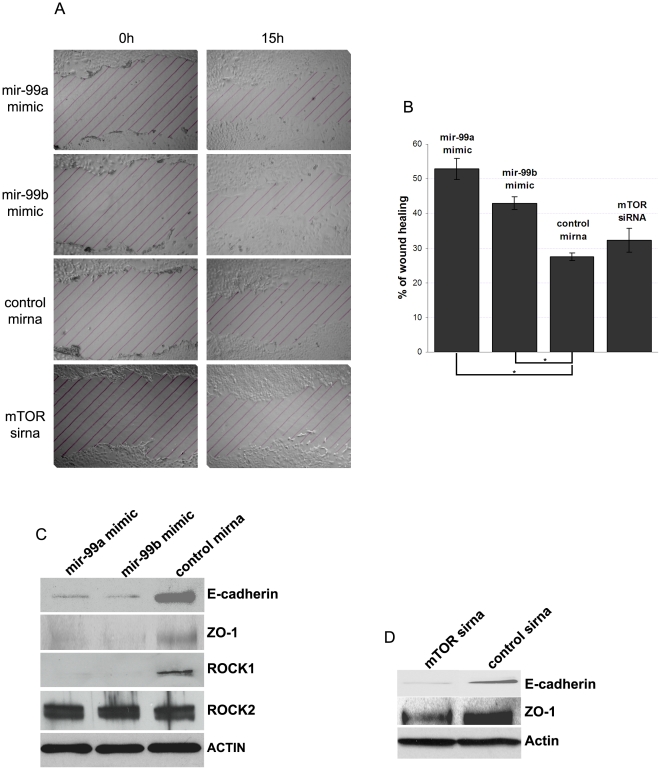
Mir-99a and mir-99b over-expression increases migration of epithelial phase NMUMG cells. (A–B) Over-expression of mir-99a and mir-99b increases the migration of epithelial phase NMUMG cells (* = p<0.05). (C) Mir-99a and mir-99b decreases E-cadherin, ZO-1 and Rock-1 protein expression level. (E) mTOR knockdown with sirna decreases expression of E-cadherin and ZO-1.

**Figure 6:**
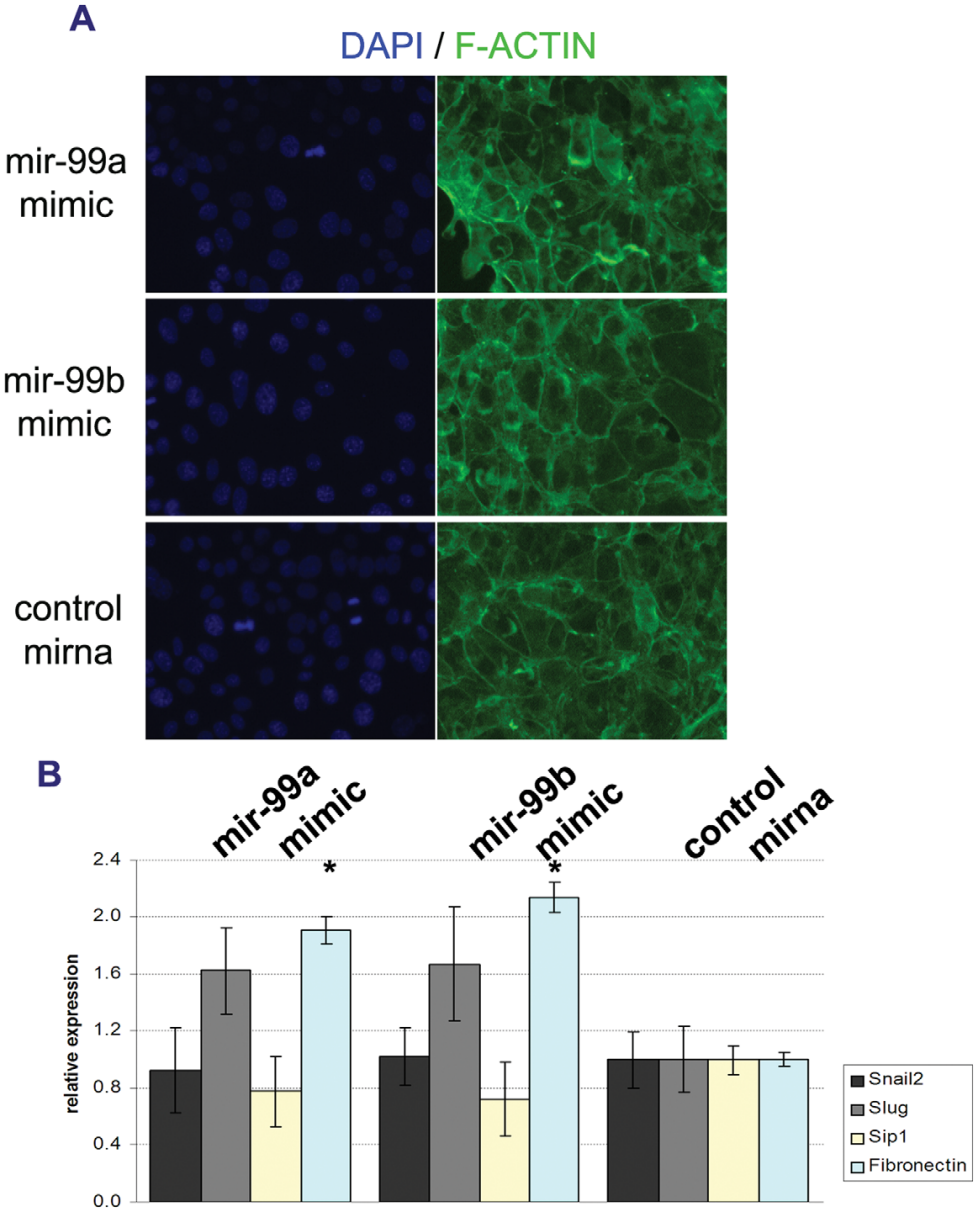
Mir-99a and mir-99b over-expression in epithelial NMUMG cells does not induce EMT. (A) F-actin staining of epithelial NMUMG cells shows that actin filaments do not change when mir-99a and mir-99b are over-expressed, indicating that NMUMG cells are still epithelial. (B) Real time PCR quantification of the transcriptional factors snail2, slug and sip1, showing no change of their expression, while over-expression of mir-99a and mir-99b induces over-expression of fibronectin (* = p<0.05).

NMUMG migration was markedly increased by mir-99a and mir-99b over-expression (Figure 5, B) which also resulted in down-regulation of E-Cadherin and ZO-1 proteins (Figure 5D). In contrast, actin distribution was not affected by mir-99a and mir-99b over-expression (Figure 6A), as shown by concentrated actin expression pattern at the epithelial junction, indicating that NMUMG cells did not undergo EMT. Moreover, the expression of known EMT markers Snail, Slug and Sip1 did not significantly increase (Figure 6B). Interestingly we found that mir-99a and mir-99b over-expression in epithelial phase NMUMG cells caused an increase of the fibronectin expression (Figure 6B), which may explain the increased migration observed in Figure 5A.

In addition, neither SMAD3 phosphorylation (Figure S2A) nor TGF-β pathway activity (Figure S2B) was affected by mir-99a and mir-99b over-expression either with or without TGF-β, suggesting that mir-99a and mir-99b over-expression increased epithelial NMUMG cells migration in a SMAD3-independent manner.

Taken together, these results suggest that while mir-99a and mir-99b over-expression was not sufficient to induce completion of EMT in epithelial phase NMUMG cells, it induced some molecular and behavioral changes which are typical of partial EMT.

### Mir-99a and mir-99b effects on proliferation and cell cycle in NMUMG cells was epithelial or mesenchymal phase-dependent

**Figure 7:**
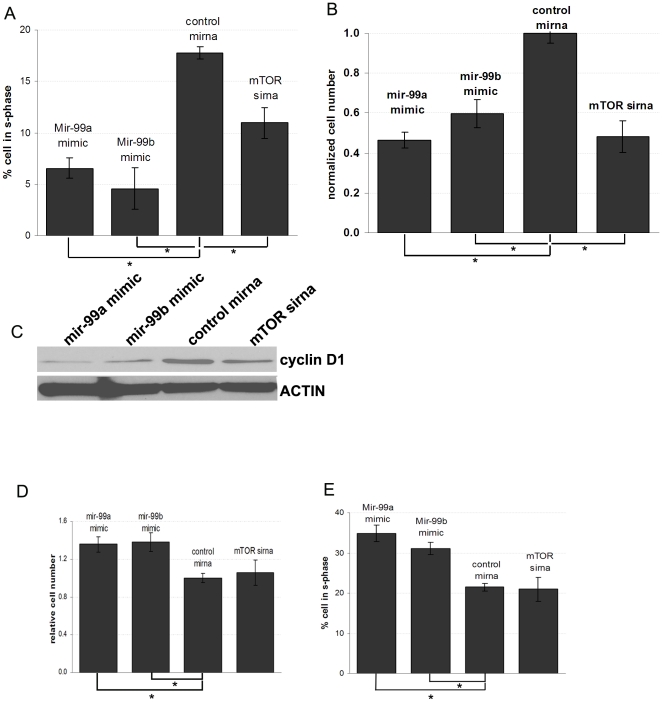
Mir-99a and mir-99b effects on proliferation and cell cycle in NMUMG cells is epithelial or mesenchymal phase-dependent. (A,B,C) Either over-expression of mir-99a and mir-99b or mTOR knock-down in mesenchymal phase NMUMG cells decreases cell proliferation. (D,E) In epithelial phase NMUMG cells, mir-99a and mir-99b over-expression increases cell proliferation (* = p<0.05).

Next, we determined the role of mir-99a and mir-99b on cell cycle and proliferation in epithelial versus mesenchymal phase NMUMG cells. When cells were cultured with TGF-β (and thus are in the mesenchymal phase) mir-99a and mir-99b over-expression resulted in decreased cell proliferation, reduction of cells in s-phase as well as reduced cyclin D1 expression (Figure 7A,B). On the other hand in epithelial phase NMUMG cells (cultured without TGF-β) mir-99a and mir-99b over-expression stimulated cell proliferation (Figure 7D,E). These data suggest that mir-99a and mir-99b over-expression affected cell proliferation and cell cycle of NMUMG cells in a phase-dependent manner, depending on whether cells are in the epithelial versus mesenchymal phase.

### Mir-99a and mir-99b induced SM-22-α expression in mesenchymal phase NMUMG cells by targetingt mTOR gene

**Figure 8:**
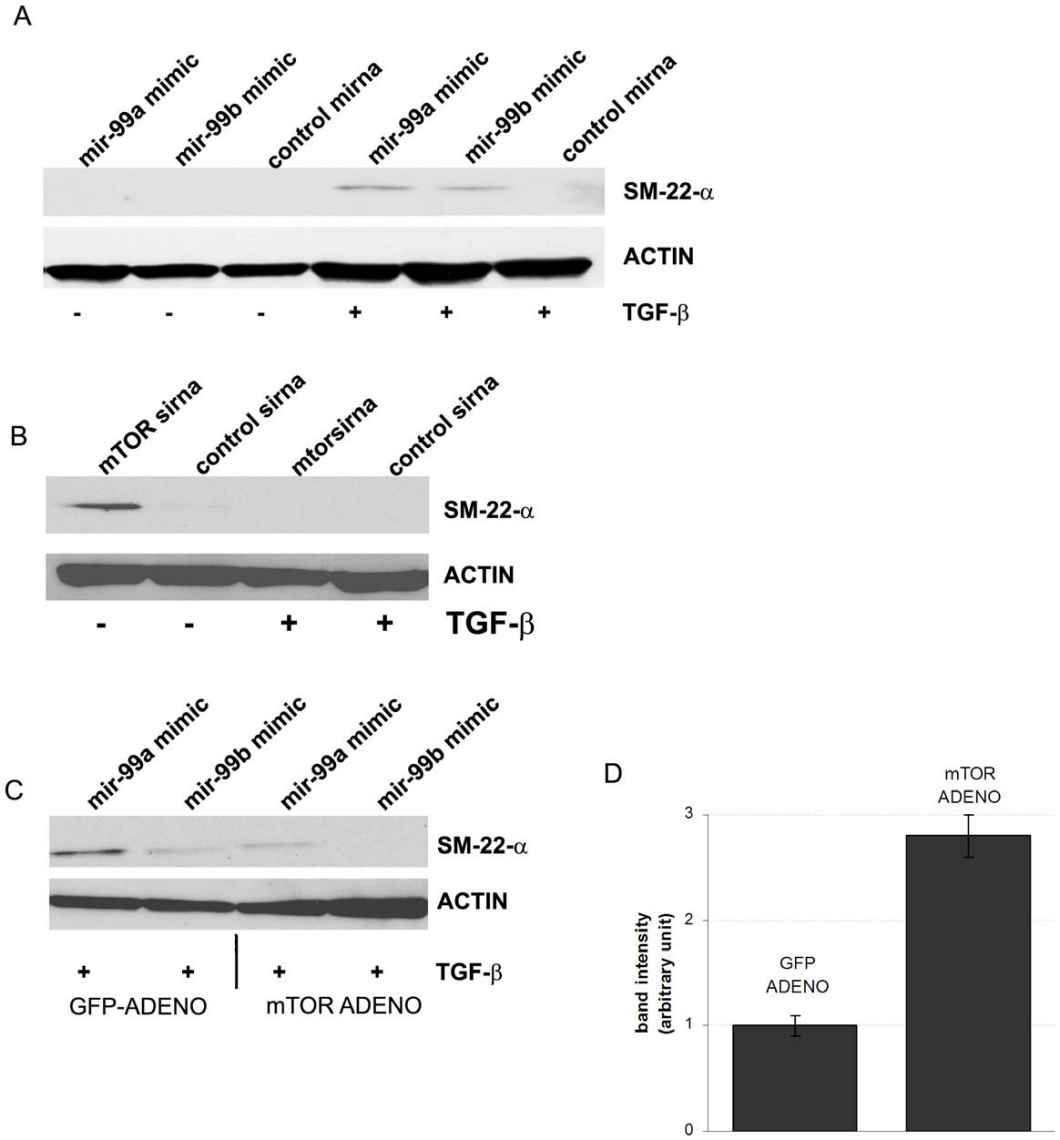
Mir-99a and mir-99b over-expression in mesenchymal phase NMUMG cells. (A) Over-expression of mir-99a and mir-99b in mesenchymal phase NMUMG induces expression of SM-22-α. (B,C) Over-expression of mTOR in mir-99a and mir-99b treated mesenchymal phase NMUMG inhibited SM-22-α expression. (D) mTOR protein expression intensity quantified by western blot to confirm adenoviral mediated mTOR over-expression.

Having determined that either mir-99a and mir-99b over-expression or mTOR knock-down resulted in inhibition of mesenchymal phase NMUMG cell proliferation, we next determined whether mir-99a and mir-99b stimulated the differentiation of mesenchymal phase NMUMG cells into smooth muscle cells. Mir-99a and mir-99b over-expression in NMUMG cells induced the expression of a marker for mature smooth muscle cells, transgelin (SM-22-α), (Figure 8A) [33] by targeting mTOR gene (Figure 8B). Conversely, SM-22-α expression was inhibited by the over-expression of mTOR in mesenchymal phase NMUMG cells over-expressing mir-99a and mir-99b (Figure 8C). Taken together, these data suggest that mir-99a and mir-99b could induce mesenchymal phase NMUMG to fully differentiate into smooth muscle cells by targeting the mTOR gene.

### Identification of mir-99a and mir-99b targets

**Figure 9:**
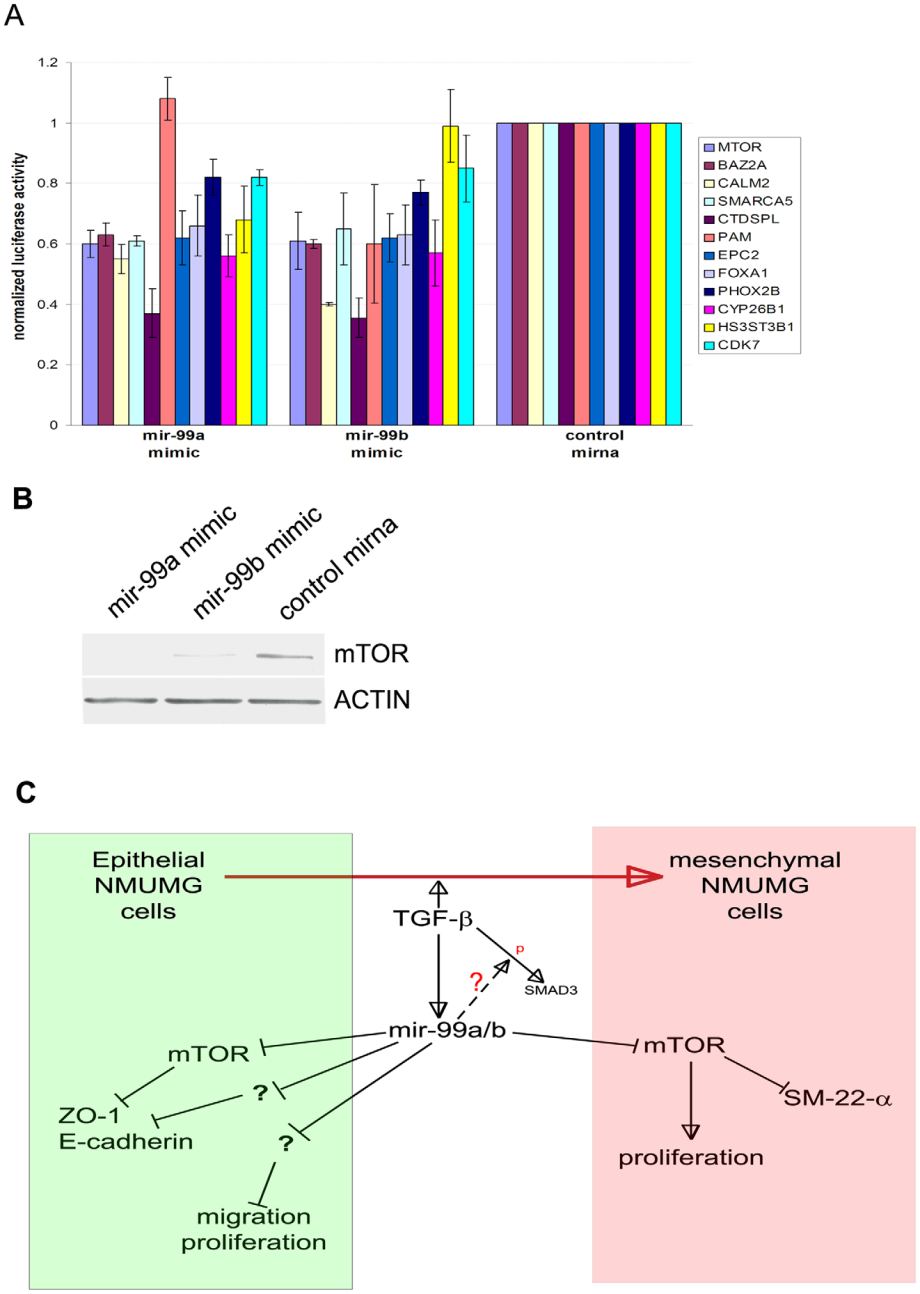
Mir-99a and mir-99b targets validation. (A) Luciferase test is used to validate most of the hypothetical targets. (B) Mir-99a and mir-99b over-expression effectively decreased mTOR protein expression. (C) Mir-99a and mir-99b working hypothesis diagram.

Micrornas target multiple genes simultaneously by binding to the 3′UTR portion of specific target mRNAs [34], [35]. The seed sequence of a microrna is responsible for mirna target identification and is only 6–8 nucleotides long, thus micrornas can potentially target hundreds if not thousands of messenger RNAs. The biological activity of most mirnas results, therefore, from the coordinated inhibition of different mRNA targets. In order to fully understand the complete array of biological activities of mirnas it is necessary to identify most of the mirna targets in our system. Mir-99a and mir-99b have a unique seed sequence, and the number of their predicted targets is quite small: 30 according to PicTar software, http://pictar.mdc-berlin.de, 38 according to TargetScan software, http://www.targetscan.org. We used a luciferase assay to validate most of the putative mir-99a and mir-99b targets (Figure 9A). We further validated mTOR gene as a target of mir-99a and mir-99b by using Western blot approach (Figure 9B). Furthermore, using a specific sirna against mTOR gene we were able to replicate many of the effects of the mir-99a and mir-99b over-expression, such as E-cadherin and ZO-1 down-regulation, in epithelial phase NMUMG cells (Figure 5E). In contrast, mTOR down-regulation per se did not affect proliferation (Figure 7D,E) and migration (Figure 5A,B) of epithelial phase NMUMG cells [28]. However, in mesenchymal phase NMUMG cells, mTOR down-regulation affected cell proliferation and cell cycle concomitantly with the observed up-regulation of mir-99a and mir-99b (Figure 6A,B,C).

## Discussion

Since EMT plays a critical role in normal biological processes and disease, understanding both its mechanisms and molecular regulators/modulators is of general biological interest. One of the well documented EMT inducing cytokine is TGF-β. TGF-β signals through both SMAD-dependent and SMAD-independent pathways in order to induce epithelial cells to become mesenchymal cells, with characteristically increased migratory and metastatic phenotypes. Many positive and negative modulators of the TGF-β pathway have been described. For instance, TGF-β signals through mTOR in a SMAD-independent way and in turn mTOR negatively affects TGF-β signaling by inhibiting SMAD3 phosphorylation.

The discovery of mirnas as a new class of gene expression regulators has further increased the complexity of regulatory networks in cell signaling biology. TGF-β regulation of mirnas expression and activity has been documented [36] while the role of particular mirnas on the TGF-β pathway is currently under study [8]. For instance, the mir-200 family of micrornas is down-regulated during TGF-β induced EMT in NMUMG cells; while over-expression of these micrornas inhibites EMT [21]. Moreover, Martello et al. has determined that mir-103/107 induces EMT in mammary gland cells by down-regulating DICER gene expression [12].

Herein, we identified mir-99a and mir-99b as two new mirnas that function as downstream regulators in the TGF-β pathway, based upon the observation that TGF-β increased the expression of both mir-99a and mir-99b in NMUMG cells undergoing EMT. However, we could not establish whether mir-99a and mir-99b are directly regulated by TGF-β, because the mir-99a and mir-99b promoters have yet to be characterized.

To determine the role of mir-99a and mir-99b in TGF-β signaling and EMT processes we inhibited mir-99a and mir-99b activity with LNA antisense probes or conversely increased their expression in NMUMG cells. We found that mir-99a and mir-99b are important components of the TGF-β pathway because inhibition of mir-99a and mir-99b negatively affected TGF-β pathway in NMUMG cells as evidenced by a luciferase activity assay. In addition, TGF-β induced migration and adhesion of NMUMG cells were inhibited upon inhibition of mir-99a and mir-99b, which further confirms that mir-99a and mir-99b activity is necessary for normal TGF-β signaling in mammary gland cells. We used human breast cancer cells 4T1 to validate that mir-99a and mir-99b inhibition negatively affected TGF-β signaling and cell wound-healing abilities (Figure S1A,B). Our data suggest that mir-99b and mir-99b may affect TGF-β signaling pathway by altering the phosphorylation of SMAD3.

Mir-99a and mir-99b blockade resulted in inhibition of TGF-β pathway activity (Figure 2A) and decreased SMAD3 phosphorylation (Figure 3A,B). On the other hand, when mir-99a and mir-99b were up-regulated, TGF-β signaling pathway and SMAD3 phosphorylation were not altered (Figure S2A,B). This contradiction can be explained by assuming that mir-99a and mir-99b have strong binding affinities for the mRNA gene targets which then alter TGF-β activity and SMAD3 phosphorylation. In this scenario most available mir-99a and mir-99b binding sites would be already “saturated”, thus the TGF-β signaling pathway would be affected only upon by inhibition of mir-99a and mir-99b, and de-saturation of the mirnas binding sites

Currently, the target of mir-99a and mir-99b that is responsible for altered SMAD3 phosphorylation is still unknown, and it is unlikely that only one target for mir-99a and mir-99b target is responsible for the altered SMAD3 phosphorylation. Several mir-99a and mir-99b targets are known to affect TGF-β signaling pathway, including mTOR, CTDSPL, CALM, PAM and FOXA1. Thus, multiple mir-99a and mir-99b targets are likely to impinge on the TGF-β pathway and be responsible for decreased SMAD3 phosphorylation when mir-99a and mir-99b are inhibited.

It is also noteworthy that, although mir-99a and mir-99b inhibition altered TGF-β signaling, it did not fully inhibit EMT progression in of NMUMG cells.

Interestingly, in epithelial NMUMG cells mir-99a and mir-99b over-expression increased migration, as evidenced by down-regulation of E-cadherin and ZO-1 and increased fibronectin expression. However NMUMG cells remained epithelial as indicated by undetectable changes of both F-actin staining pattern and expression levels transcriptional factors involved in the EMT process, such as snail1, slug and sip1. We, therefore speculate that the down-regulation of E-cadherin and ZO-1 resulting from over-expression of mir-99a and mir-99b is probably caused through mTOR, because mTOR is a target of mir-99a and mir-99b, and its down-regulation decreases E-cadherin and ZO-1 expression in epithelial phase NMUMG cells (Figure 5D). Moreover Bieri et al. have identified mTOR as a critical regulator of VE-Cadherin expression in HUVEC cells [37]. Unexpectedly, we found that mTOR down-regulation alone was not sufficient to increase epithelial NMUMG migration as also previously reported by others [28]. Thus, we concluded that mir-99a and mir-99b must be targeting other genes, in addition to mTOR, to promote migration of epithelial phase NMUMG cells.

Herein, we found that mir-99a and mir-99b affect cell proliferation in a cell phase dependent manner, depending on whether NMUMG cells are epithelial or mesenchymal. When NMUMG are epithelial, mir-99a and mir-99b stimulate cell proliferate; while they inhibit cell proliferation in mesenchymal NMUMG cells. By comparing cell proliferation data obtained after mir-99a and mir-99b over-expression and sirna induced mTOR knock-down, we concluded that mTOR gene is likely the principal target of mir-99a and mir-99b when culturing NMUMG with TGF-β (and therefore mesenchymal cells). This conclusion is confirmed by the observation that over-expression of mir-99a and mir-99b mimicked many effects of mTOR knock-down with a specific sirna. In contrast, in epithelial phase NMUMG cells, mir-99a and mir-99b are targeting at least one gene other than mTOR, whose inhibition is likely responsible for the increased migration and proliferation of epithelial NMUMG cells [28]. We have validated most of the hypothetical mir-99a and mir-99b target genes identified in silico (Figure 9A), and inhibition of some of those target genes in epithelial phase NMUMG cells may result in the increased cell proliferation and migration that we observed in our experiments.

Finally, we found that either mTOR down-regulation or mir-99a and mir-99b over-expression, in mesenchymal NMUMG cells resulted in increased expression of SM-22-α (transgelin), a marker of fully differentiated and contractile smooth muscle cells. Conversely, over-expression of mTOR gene resulted in down-regulation of SM-22-α expression, suggesting that mTOR also directly regulate SM-22-α expression in mesenchymal NMUMG cells. Consistent with our data, mTOR has been previously identified as a key gene involved in smooth muscle cell differentiation [38].

In conclusion, we have identified mir-99a and mir-99b as two novel effectors of the TGF-β pathway during EMT in NMUMG cells. Mir-99a and mir-99a are, in turn, positive modulators of the TGF-β pathway itself and also regulators of cell proliferation and migration of both epithelial and mesenchymal phase NMUMG cells. We speculate that, mir-99a and mir-99b may play key roles in cancer development and progression, as well as in embryonic development by modulating TGF-β signaling. In addition, the identification of mTOR gene as a notable target of mir-99a and mir-99b opens the door to the possibility of therapeutic applications for mir-99a and mir-99b in cancer-like diseases such as Lymphangiomyelomatosis.

## Supporting Information

Figure S1 **Mir-99a and mir-99b blockade inhibits TGF-β induced migration of 4T1 cells.** (A, B) Blockade of mir-99a and mir-99b inhibits TGF-β induced migration of 4T1 cells migration in a wound healing assay.
(TIF)

Figure S2 **Mir-99a and mir-99b over-expression does not affect TGF-β pathway.** (A) NMUMG cells over-expression mir-99a, mir-99b or a control mirna are pulsed with TGF-β for 1 h. SMAD3 phosphorylation is quantified by Western blot. (B) TGF-β pathway activity is quantified using 3TP-lux plasmid assay. NMUMG cells over-expressing mir-99a, mir-99b or a control mirna do not show changes the TGF-β pathway activity.
(TIF)
