# Supplementary figures and images for: MIR-99a and MIR-99b Modulate TGF-β Induced Epithelial to Mesenchymal Plasticity in Normal Murine Mammary Gland Cells
Source: PLoS One. 2012 Jan 27;7(1):e31032. doi: 10.1371/journal.pone.0031032 (PMC3267767; doi:10.1371/journal.pone.0031032)

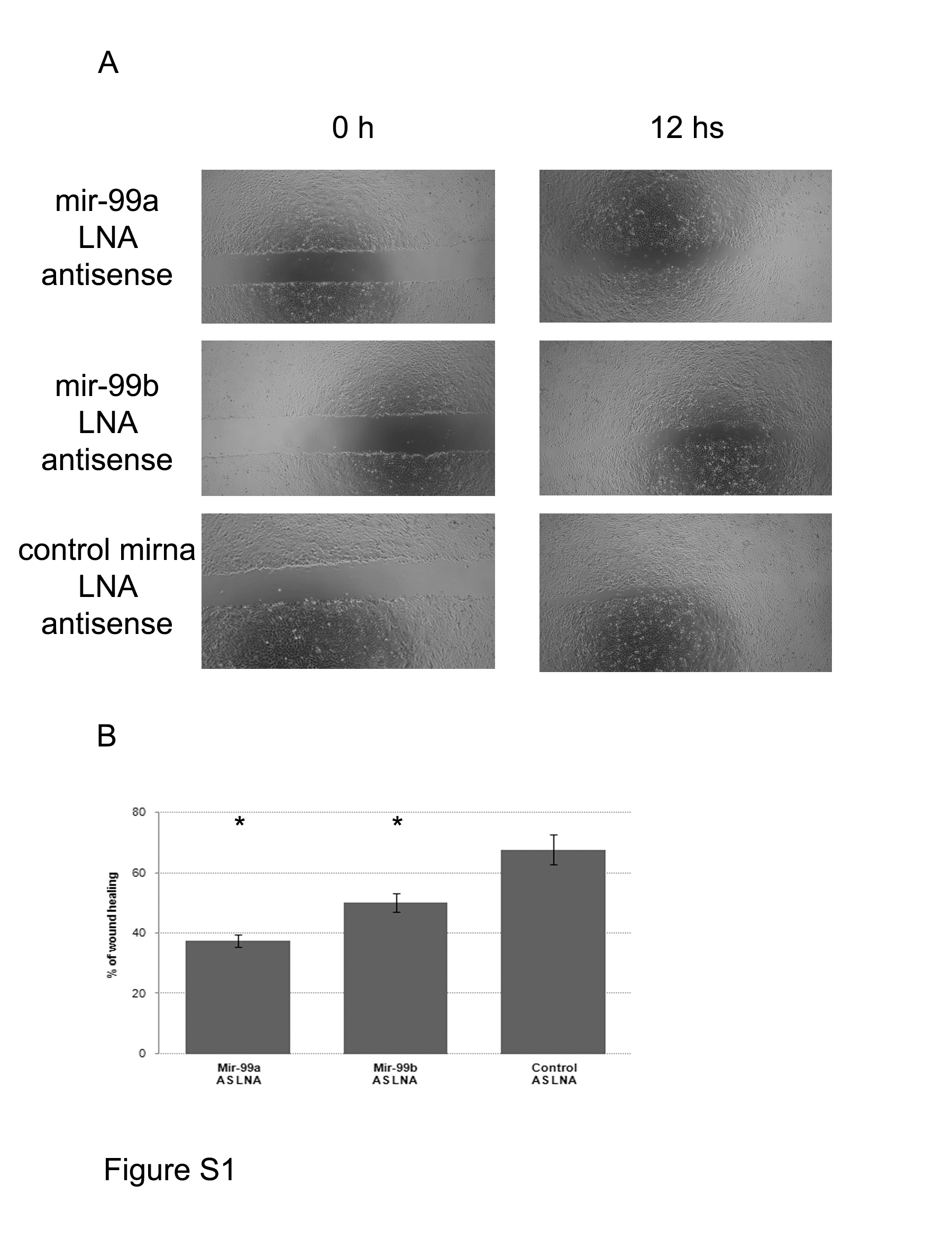

Supplement: Figure S1 — Mir-99a and mir-99b blockade inhibits TGF-β induced migration of 4T1 cells. (A, B) Blockade of mir-99a and mir-99b inhibits TGF-β induced migration of 4T1 cells migration in a wound healing assay. (TIF) [file pone.0031032.s001.tif]

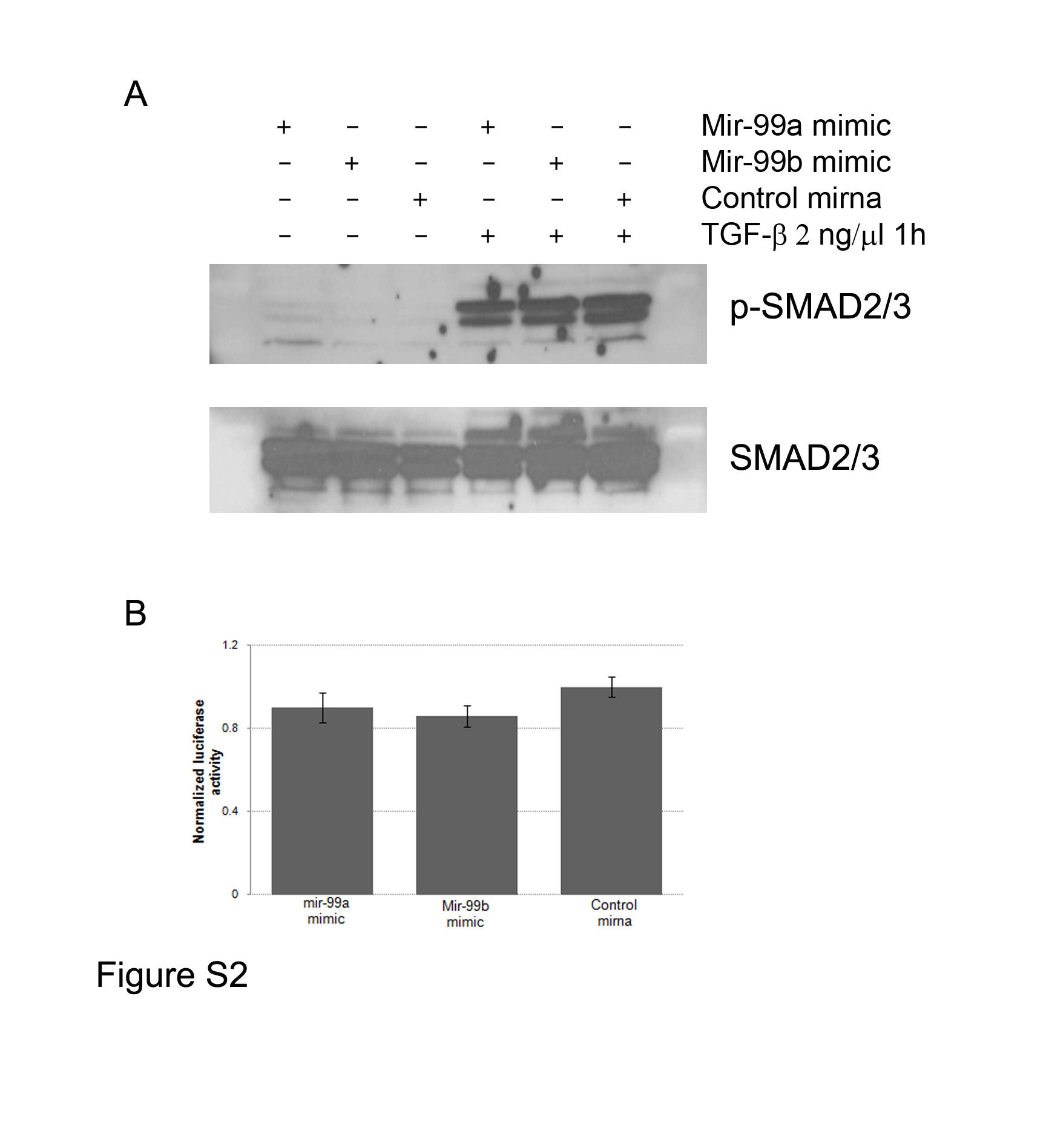

Supplement: Figure S2 — Mir-99a and mir-99b over-expression does not affect TGF-β pathway. (A) NMUMG cells over-expression mir-99a, mir-99b or a control mirna are pulsed with TGF-β for 1 h. SMAD3 phosphorylation is quantified by Western blot. (B) TGF-β pathway activity is quantified using 3TP-lux plasmid assay. NMUMG cells over-expressing mir-99a, mir-99b or a control mirna do not show changes the TGF-β pathway activity. (TIF) [file pone.0031032.s002.tif]
